# Arrowhead Technology for Digitalization and Automation Solution: Smart Cities and Smart Agriculture

**DOI:** 10.3390/s20051464

**Published:** 2020-03-06

**Authors:** Ioana Marcu, George Suciu, Cristina Bălăceanu, Alexandru Vulpe, Ana-Maria Drăgulinescu

**Affiliations:** 1 Electronics, Telecommunication and Information Technology Faculty, University Politehnica of Bucharest, 061071 Bucharest, Romania; alex.vulpe@radio.pub.ro (A.V.); ana.dragulinescu@upb.ro (A.-M.D.); 2 R&D Department, BEIA Consult International, 010158 Bucharest, Romania; george@beia.ro (G.S.); cristina.balaceanu@beia.ro (C.B.)

**Keywords:** Arrowhead technology, Arrowhead framework, IoT environment, smart cities, smart agriculture

## Abstract

The Internet of Things (IoT) concept has met requirements for security and reliability in domains like automotive industry, food industry, as well as precision agriculture. Furthermore, System of Systems (SoS) expands the use of local clouds for the evolution of integration and communication technologies. SoS devices need to ensure Quality of Service (QoS) capabilities including service-oriented management and different QoS characteristics monitoring. Smart applications depend on information quality since they are driven by processes which require communication robustness and enough bandwidth. Interconnectivity and interoperability facilities among different smart devices can be achieved using Arrowhead Framework technology via its core systems and services. Arrowhead Framework is targeting smart IoT devices with wide applicability areas including smart building, smart energy, smart cities, smart agriculture, etc. The advantages of Arrowhead Framework can be underlined by parameters such as transmission speed, latency, security, etc. This paper presents a survey of Arrowhead Framework in IoT/SoS dedicated architectures for smart cities and smart agriculture developed around smart cities, aiming to outline its significant impact on the global performances. The advantages of Arrowhead Framework technology are emphasized by analysis of several smart cities use-cases and a novel architecture for a telemetry system that will enable the use of Arrowhead technology in smart agriculture area is introduced and detailed by authors.

## 1. Introduction

Nowadays the term “smart” has multiple ramifications including “smart homes”, “smart cities”, “smart environment”, “smart agriculture”, etc. Most of them are “smart” because they benefit from IoT technology for interoperability between different communication devices with direct address to application level.

**Figure 1:**
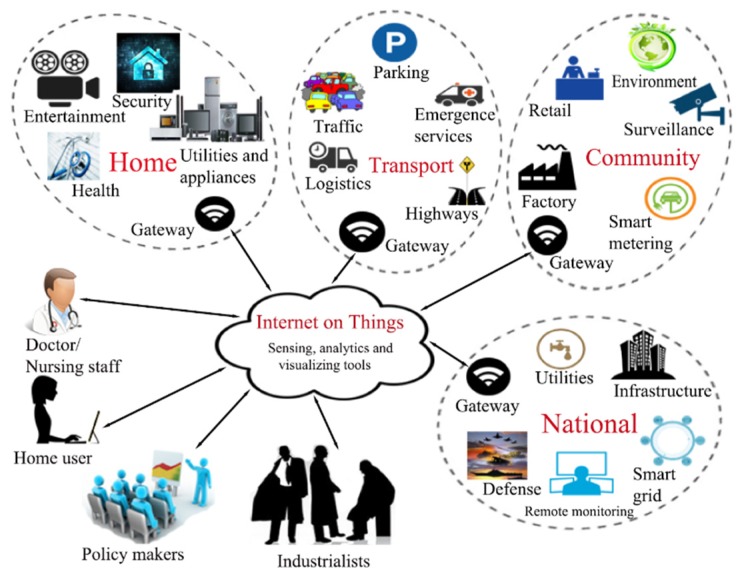
Extended applicability domains of Internet of Things (IoT) [1].

The extended applicability areas of IoT imply the use of smart devices (sensors, actuators) and IoT products such as laptops, smartphones, smart gadgets, smart vehicles, etc. interconnected to the Internet (Figure 1) [1]. The advantages of IoT devices and products include energy, time, and money decreased consumption due to efficient automation and control, Machine-to-Machine (M2M) interaction, easy maintenance. Still, the disadvantages are yet to be overcome, and they refer to increased complexity, privacy and security unsolved issues, lack of a global international compatibility standard, etc. [2].

Smart sensors-based IoT platforms have a crucial role nowadays in technological, social, and economic landscape, with practical usage in different domains such as the medical domains where even the smartphone is used in complex medical systems.

The aims of smart cities development consist in improving the urban infrastructure with minimized costs, encouraging thus innovation and upgrading the quality of life of citizens. This enhancement also includes smart healthcare that incorporates the latest smart digital IoT devices and technologies (mobile and ambient sensors, Machine Learning (ML), Artificial Intelligence (AI), etc.) to ensure progress in health activity [3,4].

For medical purposes a probabilistic framework for behavioral anomaly detection is developed in [5] based on wearable motion sensors. Although the system is tested only in a lab environment, the extension of analysis in order to upgrade the system in terms of dimensions and price is considered. Similarly, a wearable sweat sensor that can perform multiple tasks including simultaneous sweat sampling, chemical sensing, and vital-sign monitoring is implemented in [6]. The laser-engraved technology facilitates the scalability and flexibility of the sensor. Moreover, wearable sensors for table tennis stroke recognition (of five different stroke actions) are proposed in [7], based on Support Vector Machine (SVM) algorithm. The main advantage consists in ML technique use for recognition task.

Wearable electronics for healthcare monitoring lack in providing power supply solutions that can ensure stand-alone functioning within the system. Therefore, a three-dimensional cellular sensor array (3D-CSA) with a rigid structure/function symmetry that provides self-powered biomedical monitoring is depicted in [8]. The efficiency of this solution was demonstrated when measuring human heartbeat, monitoring eyeball motions, and performing active tactile imaging. This sensor advantages of low-cost, size-efficiency, adaptability, structural symmetry makes it a great candidate for future skin electronics, as well as in artificial intelligence and human computer interface areas. Still, in life-threating medical conditions such as cystic fibrosis, strokes, heart attack or cancer, customized solutions are a must [9,10,11].

For early detection and treatment estimation, authors in [9] have developed a citrate-derived synthesis platform for new fluorescence sensors with high selectivity for chloride. Thus, a wearable device (smartphone-based chloride sensor) was designed to optimize the efficiency of chloride identification in sweat for early cystic fibrosis detection. For heart attack prevention/detection a heartbeat sensor is used to check the heartbeat reading and oxygen level and further send these data through a WiFi module [10]. Although several solutions for heart attack prevention are identified, such as Garmin or Fit Bit, they lack in providing accurate data in cold weather (Garmin) or in cases of immobilized patients. The current application in [10] is still under development. Yet, for early cancer detection nanobiosensing sensors are indicated due their high efficiency [11]. Authors in [11] provide a comprehensive study on nanobiosensors including signal transduction-based nanobiosensors and biorecognition-based nanobiosensors.

Multiple solutions for the medical field using smartphones are given in [12,13,14,15,16,17,18]. Thus, the design of a smartphone-based Point-of-Care Testing (POCT) [12] represents an alternative to a mobile lab for analysis testing and has multiple advantages due to its portable feature. Several samples including blood, sweat, saliva, and tears are processed using “colorimetric, fluorescent, brightfield, and electrochemical methods” [12]. The POCT wearable device is a combination among different technologies such as the detection of using paper-based sensors (devices that are frequently used in disease diagnosis [19]), along with flexible sensors, and microfluidics. To illustrate this, test results depending on different POCT devices regarding the sweat sample information are presented in Table 1 [12].

Another efficient and high-accurate POCT for tetracycline detection was developed using milk as a real sample [13]. The fluorescence changing from red to blue due to the presence of tetracycline can be achieved using a smartphone or a home-made portable device. Point-of-care testing devices developed in [12,13] have different hardware/software limitation and their applicability area is restricted. For a more facile classification and identification of biosensors point-of-care testing devices, in [14] authors provide a review of five types of smartphone-based microfluidic biosensor system: “Smartphone-based imaging biosensor, smartphone-based biochemical sensor, smartphone-based immune biosensor, smartphone-based hybrid biosensor with more than one sensing modality, and smartphone-based molecular sensor”. Among the still-existing challenges there are: Reduced-in-size sensors may not be as accurate and sensitive as desired; expensive and complex integrated chips are yet irreplaceable; accessories attached to the smartphone may directly influence the accuracy of the systems [14]. For POCT smartphones a custom application is presented in [15] and it contains different analytical procedures. The design of custom smart sensors and systems for health care consider not only life-threatening diseases but also medical conditions that produce discomfort to patients. One approach on obstructive sleep apnea-hypopnea syndrome is given in [20] and authors designed “a textile-based wireless biomonitoring system for self-powered personalized health care” using Internet-connected clothing/textiles. The system is based on a “textile-based sensor (TS)” with accurate results when testing on elderly and weak people and its utility resides in the assessment and diagnosis of health issues.

Sensor-based IoT monitoring systems cover a large area of applicability from environmental monitoring [21], energy harvesting for IoT applications using Near-Field Communication (NFC) sensors [22] or “a linear-to-rotary hybrid nanogenerator for high-performance wearable biomechanical” [23], and in industrial equipment using wear sensor which can be 3D printed directly into the spiral using conductive material [24].

The expansion of smart cities and smart agriculture concepts takes advantage of IoT implementation together with the corresponding platforms and protocols including DIMMER platform [25], FLEXMETER platform [26], CISCO Kinetic for Cities [27] for smart cities, and Libelium/ADCON platforms [28,29] or Kaa open-source IoT Platform [30] for smart agriculture.

At a large scale, IoT systems form heterogeneous Systems-of-Systems (SoS) which describe the large-scale reconciliation of numerous free independent IT frameworks to fulfill worldwide needs from residents, consider multisystem demands. The world becomes progressively interconnected, and the SoS depend on the European riches, security, and social welfare. Since they can become unpredictable, the need to oversee SoS turns into an earnest need for European culture. In addition, SoS have an eminent assessed served market measurement in different application regions such as crisis reaction, air traffic control, water management, roadway control, etc. [31,32].

**Figure 2:**
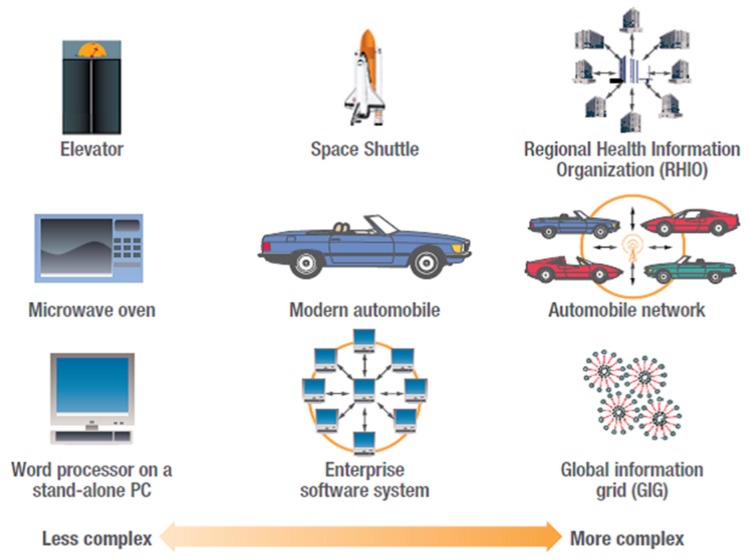
Extended applicability domains of Systems-of-Systems (SoS) [33].

Examples of SoS for applicability areas are illustrated in Figure 2 [33] and they show various areas from everyday simple systems (PCs, microwave ovens) to sophisticated systems such as “automobile networks that involve complex interactions between vehicles and the environment” [33].

Each SoS is built from several various subsystems at application level and they must fulfill requirements related to operational functioning and management. Arrowhead Framework provides support for building and interconnecting SoS based on service-oriented architecture patterns [34]. Arrowhead can be reconfigured for all types of architectures not for one or few and manages to cover and fix all security breaches that might appear in real-time applications. Following a decentralized approach, Arrowhead architecture assumes that all data are converging to one centralized node and it is already used in products worldwide.

The main features of Arrowhead Frameworks tools consist in limitless capacities, bandwidth, memory, number of devices, and is fundamentally distributed unlike all other similar existing solutions. Yet, the technology is still under development and researchers are still trying to integrate all the solutions received from engineering automation trials in different domains [35].

This paper aims to outline the advantages of Arrowhead Framework tools and in already existing IoT Framework-based architectures. Arrowhead architecture has been successfully used in several smart cities applications, smart farming and smart homes, examples that will be further reviewed. The paper is organized as follows: Section 2 illustrates the global concept of Arrowhead Framework including facilities and components; Section 3 outlines several important smart cities use-cases in which Arrowhead tools bring performances improvement (e.g., in terms of energy saving); Section 4 is dedicated to Smart Agriculture concept in Arrowhead Framework context including a novel proposed architecture for a telemetry system for precision agriculture that uses Arrowhead technology for efficiency. Section 5 provides new research lines, open issues and challenges and, finally, Section 6 concludes the paper.

## 2. The Arrowhead Framework

Cloud-based technology has a strong development in all areas, being used in various applications. The results of a study conducted on different cloud concepts are outlined in [36]. Most of the alternatives of global cloud do not fulfill the basic requirements for a local cloud:

- Automation regarding the latency of the communication and control computations;
- Scalability of automation systems that allow very large-dimensions integrated automation systems;
- Multi-party integration and speed of operations;
- Security and safety related to the automation systems;
- Simplify the process of creating applications.

A simplified representation of local Cloud Concept depicts cloud concept as a sum of multiple and sustained actions that form “global cloud” technology [36]. The concept of “local cloud” is based on the idea that certain tasks of automation, from a geographical point of view, should be encapsulated and protected. These automation tasks facilitate the creation, operation, and maintenance process, as well as system’s safety and security. The basic idea of the local cloud concept is to enable it to include the devices and systems needed to perform the desired automation tasks, thus ensuring a local “room” that can be protected from outside activities [37]. In other words, the cloud system will impose a limit to open the Internet, in order to protect the inside of the local system from what the free Internet entails.

Most applications used in smart cities or smart agriculture may require multiple cloud systems to build a large integrated automation system. Thus, the exchange of services between cloud systems should be allowed. Service exchanges between cloud systems do not ensure system delays most of the time, but properties that target security and ease of application creation are required.

### 2.1. Concepts and Functionalities

The Arrowhead Framework innovative technology depends on the idea of local clouds that comprise of SoS. Considering the Service Oriented Architecture (SOA) basics and standards, essential features are required for a local cloud:

1. Capability to register a service to the local cloud;
2. Determine which services are registered with the local cloud;
3. Enabling approximately coupled data exchange between producer and consumer systems—coordinate service exchanges;
4. Authentication of consuming systems and granting Authorization of service exchanges.

The main goal of the Arrowhead Framework architecture is to allow the development of local automation clouds considering several advantages compared to other similar architectures. These advantages derive from the fact that Arrowhead Framework architecture is based on SOA technology. Thus, there are empowered real time performance and security, combined with straightforward and low-cost building manufacturing, and at the same time, versatility through multi-cloud association is empowered. SOA technology together with the Arrowhead Framework orchestration system can lead to a service composition within a wireless network with IoT devices that minimizes the drawbacks of local cloud technology and increases the performance and robustness of the system [38].

Bringing together automatization and digitalization benefits, Arrowhead Tools enables building an open source platform for the structure and run-time engineering of IoT and SoS that meets the requirements for real-time performance, near-location devices, robust security, etc. [39].

The Arrowhead architecture consists of several systems which provide various administration. The result is an architecture from which independent automation clouds can be created. These clouds will further be capable of giving certain automation support services and provide support for bootstrapping, security, appropriate metadata, protocol, semantics straightforwardness, and for inter-cloud service exchanges [40]. Therefore, the architecture highlights three types of services by mandatory and support core systems, including application systems:

- Mandatory core services;
- Automation support core services;
- Application services.

The existence of mandatory core systems and services allows the use of a slightest local automation cloud. Mandatory core services will empower the desired fundamental properties of a local cloud.

**Figure 3:**
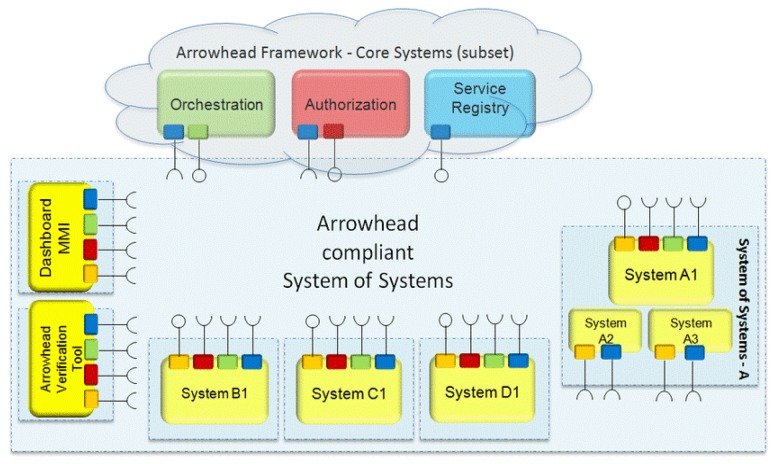
Core systems of the Arrowhead Framework [41].

Figure 3 illustrates the core systems currently defined within the Arrowhead Framework.

To build up an Arrowhead Framework local cloud three mandatory core systems are required:

1. **ServiceRegistry system**: Enables a specialist organization to distribute its administration instance(s); enables a service consumer to find (discover) what service instance(s) it is interested in consuming.
2. **Authorization system**: Enables a specialist co-op to figure out what consumer(s) to acknowledge.
3. **Orchestration system**: Enables remote control (orchestration) of which service instance(s) a consumer shall consume.

**Figure 4:**
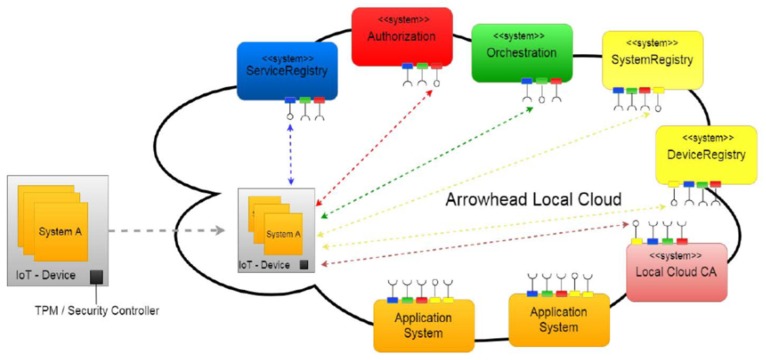
The on-boarding procedure for an IoT device: IoT system A interaction with Arrowhead local cloud [41].

Using these main core systems in addition to *SystemRegistry* and *DeviceRegistry* systems, in [41] authors analyze an on-board functioning procedure required when Arrowhead Framework interacts with a new device (Figure 4). *SystemRegistry* and *DeviceRegistry* systems offer storage for “SW-systems registered within the local cloud” and “devices registered within the local cloud” and are efficient from security reasons whenever a foreign device tries to interact with the already existing Arrowhead local cloud [41].

Use-cases for Arrowhead Framework architecture include smart medical, smart traffic (as part of smart cities), smart energy, smart farming, mobility, infrastructure, etc.

This paper aims to provide an efficient overview over the implication and methods applied for smart cities and smart agriculture when implementing the Arrowhead Framework in order to emphasize its advantages.

### 2.2. Use-Cases

According to [42], 21 use-cases involving Arrowhead Tools are currently under research in different applicability areas such as health care, energy monitoring, cloud storage, wireless communications, etc. Some of the domain have already met the requirements for Arrowhead Framework and Table 2 illustrates the correspondence between different use-cases and their current applications existing on the market.

Some eloquent use-cases (examples in Table 2) are detailed below and their efficiency and performances are described.

#### 2.2.1. SoS Engineering of IoT Devices (Including Energy Domain)

a. Energy management via Arrowhead Framework

**Figure 5:**
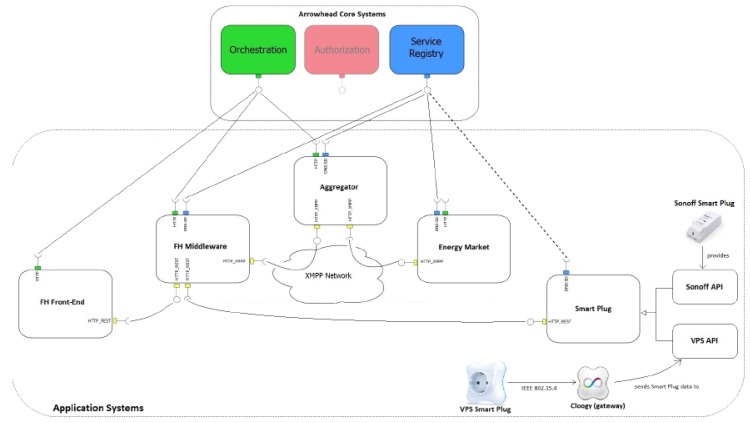
FlexHousing system with Arrowhead Framework Services [43].

**Figure 6:**
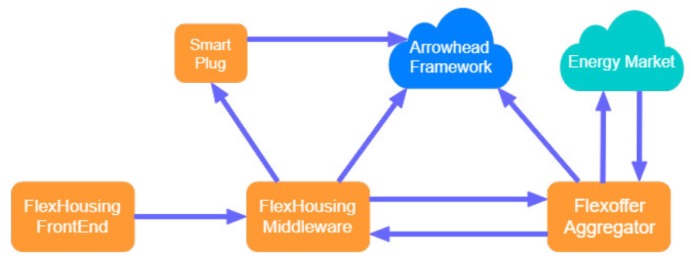
Arrowhead Framework (AF)-based FlexHousing System of Systems [43].

Engineering of IoT devices in energy domain implies the use of Arrowhead Framework (AF) in order to ensure interoperability among devices when refined energy management is a must [43]. The system addresses experiencing users with solid knowledge about energy services or markets. Thus, the FlexOffer concept used in the system is Arrowhead-compliant to facilitate the development of service oriented distributed applications. The implemented FlexHousing system uses (Figure 5):

- AF Orchestrator system: to collect data and merge systems and services; thus, they can configure protected communication channel that enable secure critical transmissions (Figure 6);

- ServiceRegistry system: for ensuring flexible and dynamic interoperability between different devices (systems). In SoS, the ServiceRegistry system uses features of service producers for publishing their specific applications and identifying different users;

- Authorization system: to control a certain service accessed by a specific user (based on a rules list).

The research in [43] was intended to extend “to different smart plugs that obey to different interaction patterns, and data regarding the energy saved in real-world deployments will be collected”.

b. Efficient energy monitoring chain

**Figure 7:**
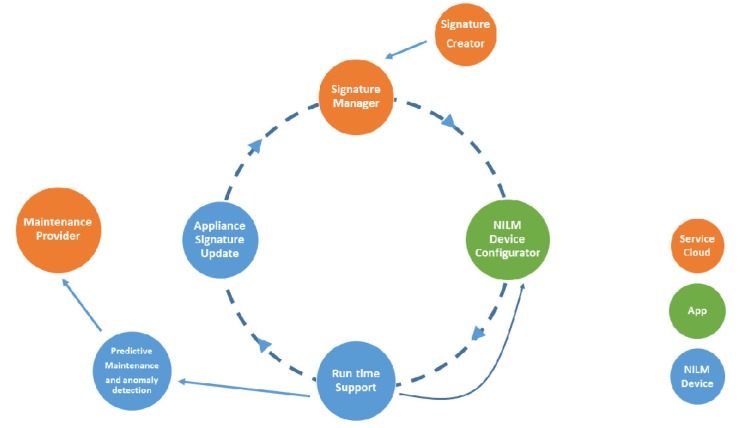
Tool ring configuration of the system [44].

The Arrowhead Framework approach in [44] assumes that the created tools will be interconnected using this new technology. Though the system is still under development, the authors introduce a Non-Intrusive Load Monitoring (NILM) method to monitor the power consumption of individuals from other consumptions at the same measurement point. The ring configuration of the implemented tools (Figure 7) consists of *Signature Creator tool* (supports the signature creation process) and *Signature Manager tool* (manages exchange of signatures).

To facilitate tools interconnection, to reduce the complexity and costs of the system, Arrowhead Framework is considered as a viable candidate for future experimental tests. It is considered that every tool will represent a compatible service offered to service providers and consumers.

#### 2.2.2. Production Support, Energy Efficiency, and Flexible Data Acquisition System

Production processes and equipment monitoring can prove to be efficient when a combination between IoT facilities, Arrowhead Framework capabilities, and Machinery Information Management Open Systems Alliance (MIMOSA) data model is used to fulfill this goal [45]. The configuration of Arrowhead Framework architecture can handle SoS requirements and adapt efficiently to dynamic changes in processes.

**Figure 8:**
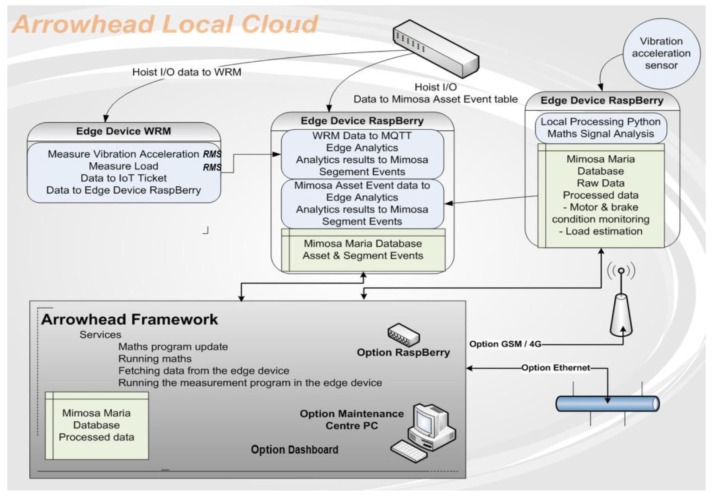
Arrowhead local cloud configuration for hoist operation monitoring [45].

A model for Arrowhead local cloud configuration for hoist operation monitoring is depicted in Figure 8. From the attached local edge devices, data is processed through the gateway to the cloud in a flexible way. At the gateway layer, process-related data are analyzed. Here, through the Service registry, Orchestration and Authorization, one can publish the application system services. Next, the consumers can subscribe to these services. The MIMOSA data model is used for the acquired data storage and can act also at edge, gateway, and cloud layers. For example, all sensory data are kept at gateway level. When necessary, the gateway posts the data to a MIMOSA database, which, further, can interact with the Cloud services and analytics.

The main advantages of Arrowhead Framework architecture identified by authors in [45] include:

- Cloud and inter-cloud approach can be managed with different security methods;
- The use of the *Service Registry, Orchestration,* and *Authorization services* in Arrowhead Framework provide efficiency in case of rapid change of SoS elements or introduction of a new system, or a redundant system takes over the service providing role from a failing one [45];
- Additional services of Arrowhead Framework can be involved in the dynamic monitoring: *Event Handling, QoS monitoring, Inter-Cloud servicing*.

**Figure 9:**
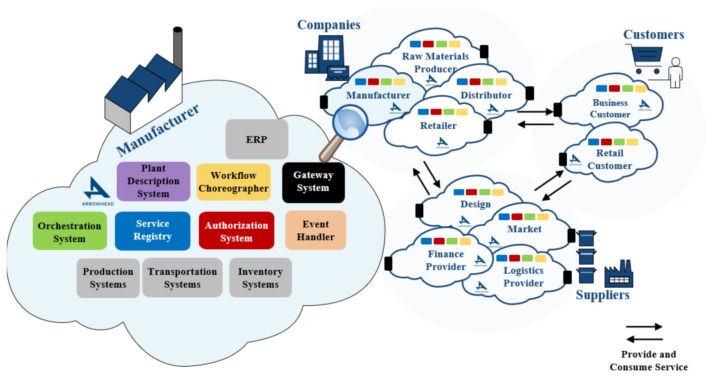
Arrowhead-based supply chain management [46].

Similarly, in [45], Arrowhead local clouds can be operated by Suppliers, Companies, and Customers in Industrial Internet of Things (IIoT) systems. Thus, by using *Orchestration System, Authorization System*, *Service Registry,* and *Event Handler* in Arrowhead Framework, a complex supply chain management process is illustrated in Figure 9.

In [46], the authors propose a use-case in Paper Production, thus demonstrating that Arrowhead Framework design enables Industrial IoT scenarios based on a service-oriented approach.

**Figure 10:**
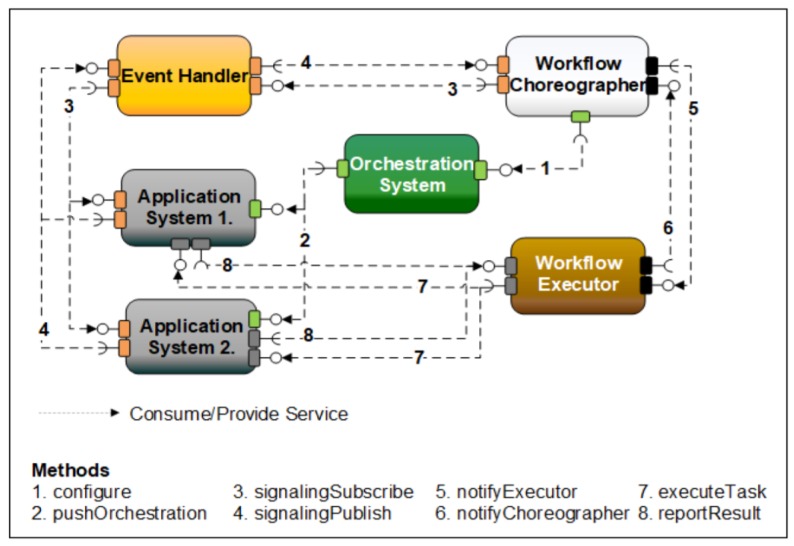
Arrowhead-based cloud interconnected with Workflow Choreographer [47].

The IIOT system is also described in [47] together with another use-case for Arrowhead Framework for data-driven workflow management. Taking advantage of Arrowhead Framework SOA-based architecture, digital production in IoT context is considered for cloud communication. From Arrowhead Framework, *Orchestration System* is used as an interface for modifying the interactions between application systems. Next, *Event Handler* sends notifications concerning the events and monitors these events over the network. The Arrowhead core systems represent the data-driven engine structure and the workflow is controlled by a Workflow Choreographer (automated workflow management engine of the Arrowhead framework) that exploits the “*Orchestration Push*” service. The information on services reservation are achieved via *Event Handler* (Figure 10).

Therefore, the Workflow Choreographer (formed as a new engine of the Arrowhead Framework) can ensure the necessary support for the IIoT System of Systems through its SOA-based principles derived from the Arrowhead Framework.

#### 2.2.3. Local Clouds Autonomous Configuration. Deployment and Configuration of SoS Systems.

Arrowhead local cloud autonomous configurations is the most common use-case developed nowadays [49,50,51,52,54,55]. The studied use-cases refer to service interaction through gateways for inter-cloud collaboration within the Arrowhead Framework [39], secure and trusted inter-cloud communications in the Arrowhead Framework [48], interacting with the Arrowhead local cloud: On-boarding procedure [50] and a multi-usable cloud service platform: A case study on improved development pace and efficiency [51].

**Figure 11:**
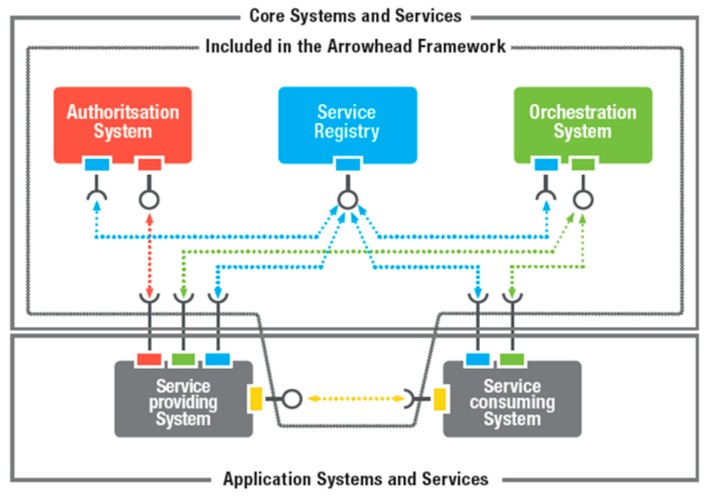
General architecture for multi-usable cloud service platform [51].

Starting from Arrowhead Framework architecture, in [51], authors have designed a multi-usable cloud service platform to be used in a micro/small/medium-sized enterprise in Sweden (Figure 11). The proposed use-case referred to optimizing the recycling management.

The results outlined that the development pace and efficiency of the enterprise have been improved by 50–75% when using the Arrowhead Framework and changing development processes/practices.

**Figure 12:**
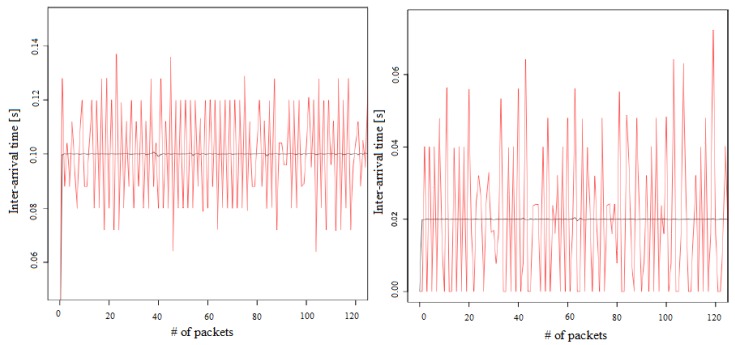
Use case (**left**): 100 ms sending period without Quality of Service (QoS) prioritization/use case (**right**): 20 ms sending period without QoS prioritization [53].

Not only criteria related to efficiency and security must be taken into consideration when using Arrowhead Framework architecture, but QoS should be a key performance parameter [53,54,55]. In [53], the performances of a Long-Term Evolution (LTE) network (with a maximum capacity of the radio cell of 60/30 mbps) in different scenarios of IIoT intra-cloud use cases are evaluated considering the model for the edge level Arrowhead Local Cloud in Arrowhead Framework and Arrowhead Core devices (provider and consumer). The LTE management tools is configured using a dedicated QoS Class Identifier (QCI) traffic class for the delay sensitive Arrowhead traffic. It was noticed that 10 ms delay in packet delay occurs when sending period of an Arrowhead application service provider (within 5 to 20 ms range) (Figure 12). This delay is unacceptable in an LTE communication because it leads to QoS degradation in IIoT use cases.

This delay achieved with an Arrowhead application service provider can be overcome by using “SDR-less LTE micro-cells, or by the upcoming 5G networking technologies” [53].

**Figure 13:**
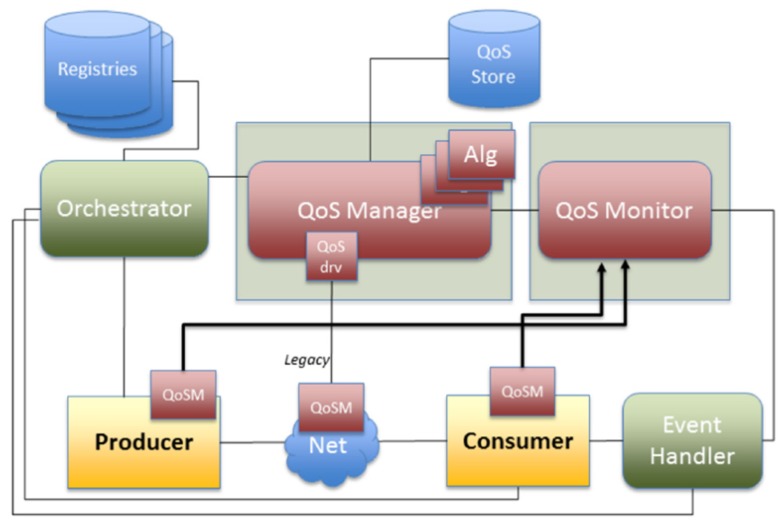
Architecture for QoS in Arrowhead [54].

According to [54], *QoSSetup* and *Monitor* are core services for QoS supporting in Arrowhead local clouds (Figure 13).

Testbed based on Flexible Time-Triggered Switched Ethernet (FTT-SE) protocol measures the time interval the consumer requires until it starts using a QoS-enabled orchestrated service, and the effect of QoS guarantees, considering that the service producer is limited to only one service. It was illustrated how QoS can be successfully applied to Arrowhead-compliant local clouds at an architecture level, at verification algorithms level, and how to configure this QoS.

Use-cases for Arrowhead Framework architecture include smart medical, smart traffic (and at a larger scale smart cities), smart energy, smart farming, mobility, infrastructure, etc.

This paper aims to provide an efficient overview over the implication and methods applied for smart cities and smart agriculture when implementing the Arrowhead Framework in order to emphasize its advantages.

## 3. Arrowhead Framework in the Landscape of Smart City Frameworks

Smart municipality [55], smart city services [56,57], utilities systems control in urban environment [58], energy optimization and next-generation building management systems [59], public safety, transportation, healthcare and education [60] are examples of applications available in a smart cities context, aimed to increase the quality of life of the citizens [61].

All these applications are disjointed without a framework that integrates them, providing interoperability for heterogeneous devices and services, being capable to gather and manipulate reliable data sources [62] in a secure way and to transform them into valuable information for citizens and other stakeholders. To achieve these, several smart city frameworks were proposed. Frequently, these frameworks envisage the importance of social factors, Information and Communications Technology (ICT), legal compliances, sustainability, as well as different factors related to economy [63]: Potential for urban development [64] as a response to better services and infrastructures [65].

### 3.1. Smart Municipality

**Figure 14:**
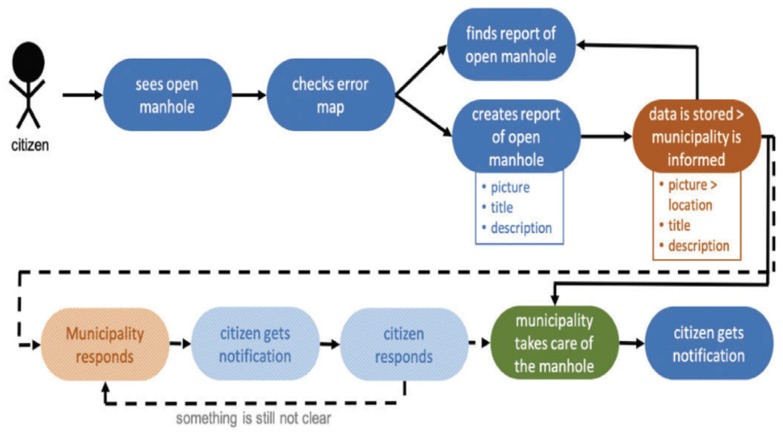
Smart municipality design [55].

In [55], an incident management system takes into consideration the use of Arrowhead architecture to provide the IoT framework for relevant data from sensors collection and to offer security, real-time communication, and safety in local cloud automation for different incidents scenarios (Figure 14).

Furthermore, the Arrowhead Framework should ensure interconnectivity between citizens and authorities’ applications in one cloud or distributed separate cloud [55].

### 3.2. Smart City Services

**Figure 15:**
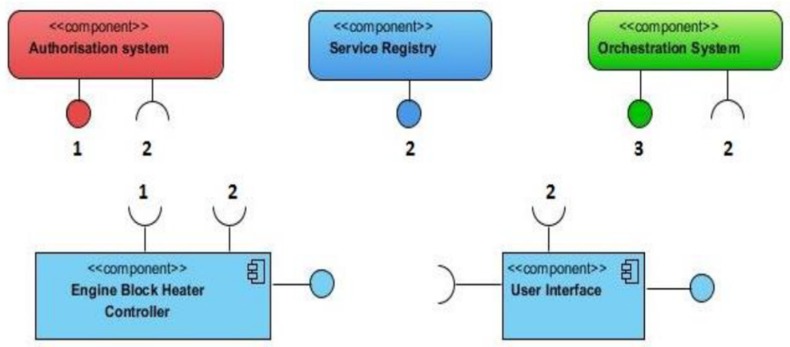
Software app for Engine Block Heater Controller based on Arrowhead Framework [56].

Arrowhead Framework architecture can be used to develop smart cities applications as illustrated in [56]. This project aims at areas such as Smart Buildings and Infrastructures, Energy Production and End-User Services, Virtual Market of Energy, etc. [56]. The authors develop pilot applications for each domain in order to demonstrate the use of Arrowhead Framework (Figure 15). Two of the implemented applications (for managing and monitoring system for streetlights and for engine block heaters) show that the dimming of streetlights can be accomplished based on the luminance and the heating time of engine block heaters based on temperature.

**Figure 16:**
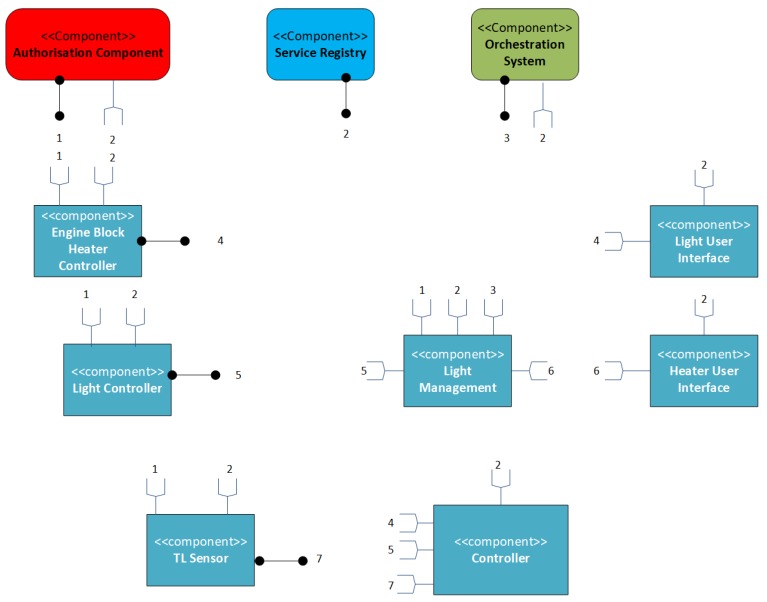
Overall system with street lighting and car heating system [56].

The final integrated system that incorporates both streetlight and car heating systems included in the Arrowhead Application is illustrated in Figure 16.

The connections between producers and consumers are outlined by numbers. The Arrowhead core services (the Controller) searches for the appropriate service producer within the Arrowhead network consuming other controllers (named application service producers) such as Engine Block Heater, Light, and TL sensors.

Aside from energy saving, the main advantage of the proposed solution in [56] by comparison with the solution in [57] consists in components reusability due to SOA technology.

**Figure 17:**
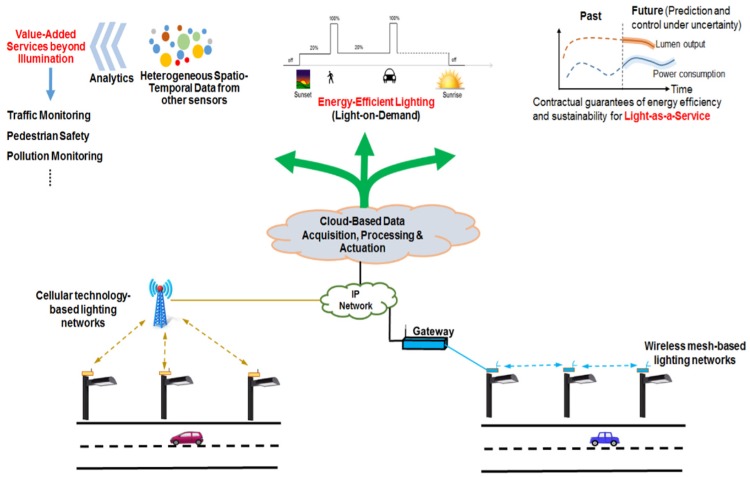
IoT solution for Lighting-Enabled Smart City Applications and Ecosystems (LENSCAPEs) framework [57].

Another IoT solution for street lighting “Lighting-Enabled Smart City Applications and Ecosystems (LENSCAPEs) framework” [57] uses light-on-demand capability based on environmental sensing for energy-saving, as well as real-time monitoring of the lighting system (Figure 17).

**Figure 18:**
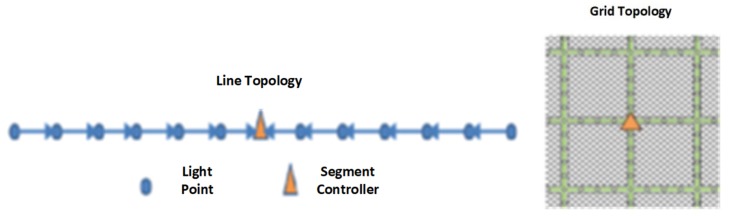
Basic topologies for IEEE 802.15.4g-based Outdoor Lighting Networks (OLNs) evaluation [57].

Two topologies (Figure 18) have been considered in [57] in order to evaluate the performances of IEEE 802.15.4g-based Outdoor Lighting Networks (OLNs) (the maximum throughput and network delivery delay given 5K bytes per pole per day. The results are given in Table 3.

From Table 3, it can be noticed that the throughput decreases as the number of poles increase (for 10,000 poles, the throughput is significantly lower: 94.53 KB per day) and, as the number of poles doubles, the throughput doubles also. As expected, the delay increases with the number of poles and decreases with the decrease of the data rates.

### 3.3. Utility Systems Control and Energy Optimization

**Figure 19:**
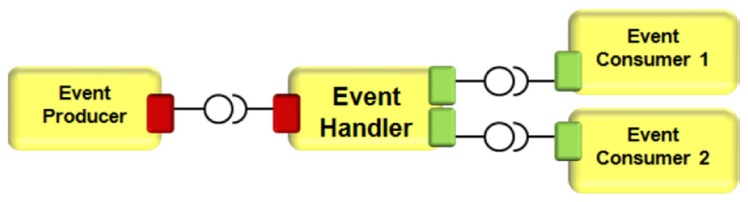
Event Handler system design [58].

Smart city approach with utilities systems control using the Event Handler core system of Arrowhead Framework is detailed in [58]. The Event Handler System is developed in a real urban environment as a Message Queuing Telemetry Transport (MQTT) service broker enabling interconnections between embedded devices such as sensors and actuators used for Smart Cities applications (cities utilities, heating, and constructions systems). The Event Handler system is depicted in Figure 19.

As depicted in Figure 19, the Event Producers send messages to different Event Consumers via Event Handler. Among the roles of Event Handler, there are storage of producers, consumers, and events, as well as events filtering. The communication protocol for Event Handler system is MQTT, a wide use protocol for IoT embedded devices used in various IoT applications such as smart agriculture, smart building, etc.

Although the Event Handler system design was successful, the authors in [58] leave room for improvement in terms of system security, costs, and complexity.

**Figure 20:**
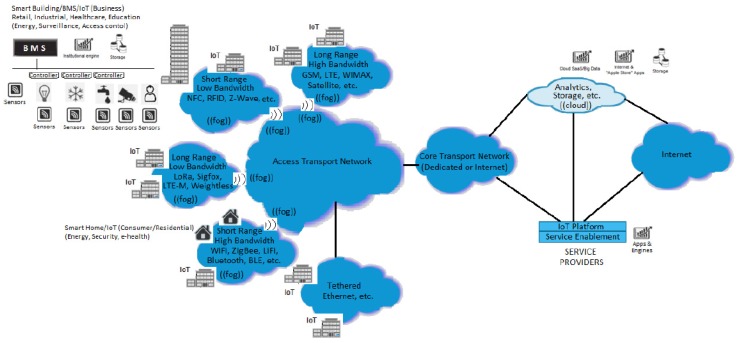
Global IoT-environment solution for smart concepts [59].

In [59], authors discuss IoT requirements and different architectures for smart building optimization from an energy point of view in smart cities. Figure 20 outlines the significant impact of IoT solutions (sensors, controllers, aggregation networks, cloud applications, etc.) over smart cities concept including smart homes, smart building, etc.

For the IoT architecture several solutions including *Arrowhead Framework, 3GPP, Industrial Internet Reference Architecture (IIRA), ISO/IEC WD 30,141 IoT reference architecture (IoT RA), Reference Architecture Model Industries 4.0 (RAMI 4.0); IEEE Standard for an Architectural Framework for the IoT* are presented in [59]:

- *Arrowhead Framework*: Incapsulates the idea of using TCP/IP everywhere. The architecture is SOA-based and ensures interconnectivity among all SOA environments. The framework was defined for smart cities (smart energy, smart homes, smart buildings, etc). The authors in [59] suggest that a possible efficient architecture may be Arrowhead architecture for ensuring data security due to its SOA-based concept.

- *IIRA*: Is a standard-based architecture targeted on functionality domains. Enhanced interoperability and technology developments and standardizations are the main purposes of this architecture.

- *IoT-RA*: Is an IoT model that enables interoperability among IoT devices with security aspects included.

- *RAMI 4.0*: Is an unified reference architecture with assets description including sensors, actuators, controllers, etc. An “Administration Shell” (AS) which stands as Data-Warehouse for the asset ensures the virtual representation of the real asset [59].

- *IEEE Standard for an Architectural Framework for the IoT*: Is a concept IoT architecture with development based on security, protection, privacy, and safety aspects.

In [62], CityPulse Framework is proposed as an adaptive framework able to gather raw smart city-related data, implementing tools for data processing and forwarding. Similar to Arrowhead Framework, CityPulse uses semantic annotation to achieve the interoperability of raw data. Moreover, both platforms are provided with device and data discovering capabilities.

SmartSantander [66] is a three-tiered SmartCity platform built on two planes: *IoT experimentation* plane and *Testbed observation and management* plane. This separation between planes enables a facile and automatic resource management.

**Figure 21:**
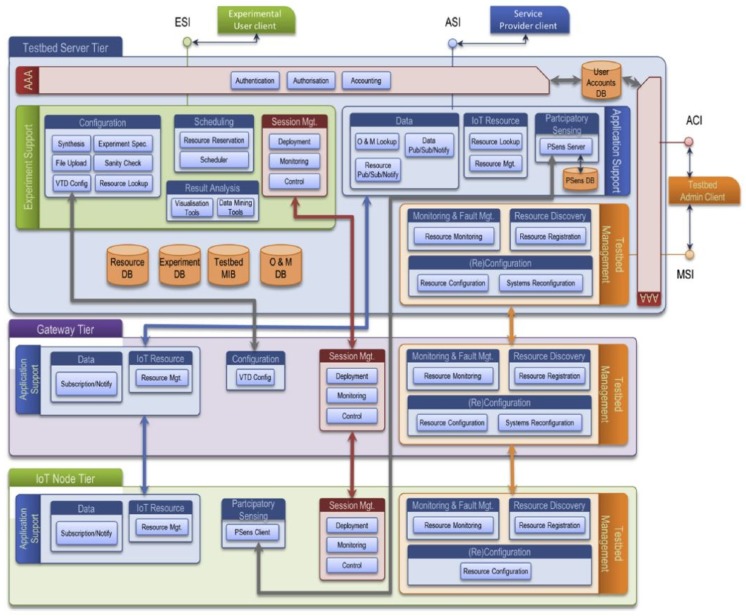
SmartSantander Smart City architecture [66].

In Figure 21, IoT node tier—comprising the IoT devices, IoT gateway—responsible for the communication between IoT devices and the core network—and server tier, in charge of data storage, as well as applications and service hosting, were depicted.

Similar to the Arrowhead Framework, SmartSantander proposed architecture uses several services and features to implement the expected functions such as *Publish, Subscription, and Notify* services to disseminate events and *Authentication, Authorisation, and Accounting* rules to enable the service provider to authorize a consumer to use a resource or to deny access.

In Arrowhead system, reconfiguration and resource configuration-related information is located in the core system, which corresponds to SmartSantander to Server Tier.

The authors in [66] mention the following use-cases, also valid for Arrowhead: Smart City: Environmental monitoring (Air quality, Waste management), Outdoor parking management, Driver Assistance, Smart Irrigation for parks and public gardens, Augmented reality (for museums, culture institutions and others), Participatory sensing (citizens devices connected to the platform feeding information to enable services dependent on the citizen). According to [67], an architecture comprising Smart Mobility features may be a key factor in reducing travel time and traffic jams.

## 4. Smart Agriculture in Arrowhead Framework Context

### 4.1. Use-Cases

In the context of continuous growing of the population in large cities, agriculture will expand its evolvement around and in big cities in order to ensure food for everybody. Agricultural activities in smart cities can be tagged as *smart agriculture* activities and, similar to other worldwide activities, they present an increased need for data security, reliable interconnection between devices and equipment, certain QoS ensured, etc.

By introducing the Arrowhead concept in agriculture, it is expected to reduce development costs and introduce flexible and secure automation solutions by 25–60% of the total implemented solutions. To facilitate the development of new applications, the basic services included in Arrowhead are installed in the analysis area, and the data transmission is secure by the Virtual Private Network (VPN).

Smart Agriculture in the context of Arrowhead platform consists in the implementation of a decision support system for in-site specific of agricultural culture. It is based on a cloud IoT end-to-end solution that can integrate various traditional and novel sensors (weather stations, Very High Resolution satellite (VHR), and Unmanned Aerial Vehicle (UAV), portable biosensors), as well as an advanced analytical engine, including cutting edge approach to artificial intelligence techniques and blockchain technologies, for the processing, modelling, and securing of the collected data and their conversion in useful knowledge through the specific intelligent applications. Corresponding web and mobile interfaces for the visualization of results and recommendations by farmers can also be generated keeping in mind an easy understanding and usability.

The objectives followed by an Arrowhead Framework design for smart agriculture include:

- Implementation and integration of a multisensory platform including;
- Development of intelligent modules and software components to establish potential correlations between collected data and other data sets available online;
- Development and validation of the IoT platform as intelligent decision support system by transferring knowledge and recommendations to farmers;
- Development of Blockchain for traceability of crops products;
- Final demonstration in an industrial environment.

**Figure 22:**
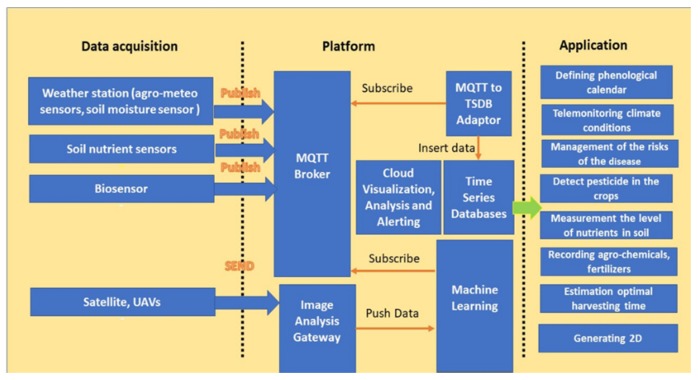
Smart Agriculture platform.

The core services of smart agriculture are included into, and shipped in the form of, Arrowhead Framework (Figure 22).

These services, through their supporting systems, take care of all the actions that do not pertain to the functional requirements of a particular use case, but are instead related to the maintenance of the local cloud itself and on the nonfunctional requirements of the use case in general.

Even in the most minimal local cloud, the core provides registration and discovery of services, systems, and devices (*ServiceDiscovery* service, or SD), security (Authentication service, or AA), and orchestration of complex services (Orchestration service, or O). The application systems are also consumers of the core services, depicted in red, green, and blue, respectively.

**Figure 23:**
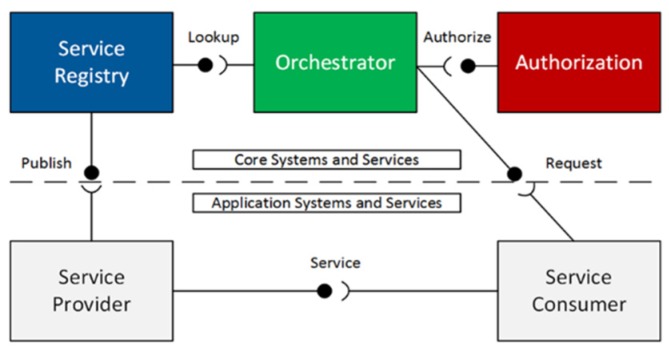
Open source system in Arrowhead Framework [20].

Open source systems are detailed in the Core services part of Arrowhead Framework (Figure 23).

Vertical farming is developed by authors in [68] and the challenges consist in data security, scalability, and IoT framework. Arrowhead Framework is proposed to register IoT devices that allow interconnectivity between heterogeneous systems and components ensuring at the same time, data security [68].

Using the parameters adjusted for scalability, safety, security, and self-adaptability of the system, authors intend to implement further a robotic appliance.

### 4.2. SmartAgro Project

SmartAgro [69] is a Romanian national precision agriculture project, that uses digitalization aspects for smart agriculture presented in [70], aspects related to global positioning systems, cloud computing, IoT, and their effects on intelligent farming. The aim of the project is to develop a telemetry system with self-reconfigurability and self-diagnosis functions that will enable the use of IoT/M2M communications in areas with limited Global System for Mobile Communications (GSM) signal.

The proposed telemetry system will be used in all types of agricultural fields (viticulture, fruit growing, vegetable growing, cereal cultivation, etc.). Among the crops for which there are standard monitoring applications, verified on tens of thousands of stations installed in the world, there can be mentioned: Vines, apple, pear, potato, wheat, corn, etc. For vines, for example, the three main diseases, manna, mildew and rot, are managed differently, by accessing software extensions that incorporate the experience of many specialists in the field.

The telemetry system also allows the measurement and storage (by dedicated sensors means) of the fundamental parameters for irrigation management, respectively the amount of precipitation, soil moisture, and evapotranspiration, as well as the remote control of irrigation systems. The system also provides other important data for precision agriculture, such as: The amount of heat received daily and the cumulation of culture (in degrees-days), the number of hours of frost/heat, daily statistics, monthly/annual for all measured parameters.

### 4.3. Proposed Smart Agriculture Architecture Based on Arrowhead Framework

**Figure 24:**
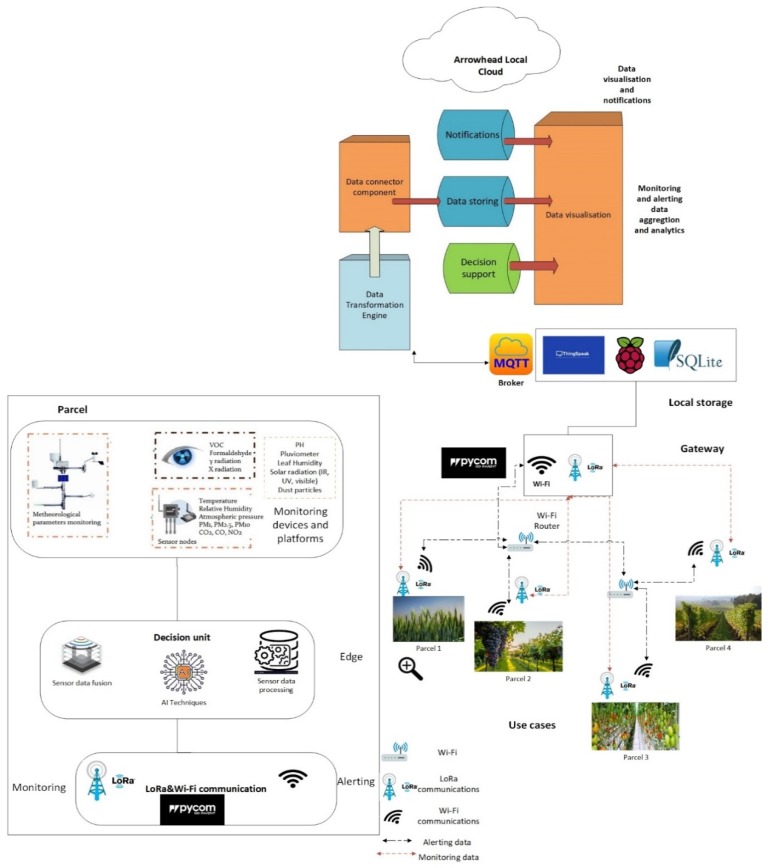
SmartAgro innovative architecture.

The proposed architecture (Figure 24) is different from the traditional architectures which use only four or five layers. Generally, the four-tier architectures include: Device layer, Network layer, Cloud layer, and Application layer.

The proposed Arrowhead Framework-based SmartAgro architecture aims to optimize the functions of the telemetry system on several layers by using the Arrowhead Framework concept of local clouds. It must be stated that the use of Arrowhead represents a novel approach in precision agriculture.

The SmartAgro architecture consists of several local clouds, each corresponding to a stakeholder in agriculture (a farm, etc.) Each local cloud consists of the following subsystems:

- **Parcel**: The parcel represents the unit element of a local cloud. It corresponds roughly to a parcel in a farm, but may sometimes represent a group of parcels, depending on the application and use case. Each parcel is composed of several layers:
  ○ **Monitoring devices and platform layer**: This layer contains all the necessary hardware and software to collect the raw data in the field and to store them for short-term: Sensors, as well as professional telemetry stations can be used.
  ○ **Edge layer**: The edge layer is where the novelty lies as it contains what the authors have termed a decisional unit based on sensor data merging and processing, as well as AI techniques used for decision support, which is likely to be extremely important in precision agriculture
  ○ **Communication layer**: This is where the communication between the parcel and the gateway happens. It is envisioned to use low-power and possible long-range technologies suitable for transmitting data with batteries that can last for years (e.g., LoRaWAN).
- **Gateway**: A logical entity, the gateway is the entry point to the SmartAgro cloud platform, lying in the middle of what we call the local cloud. The reason for a separate gateway is to leave the possibility of connecting other technology gateways besides (e.g., LoRa) and controlling the flow of information towards the local storage.
- **Local Storage**: The local storage consists in a Database Management system (DBMS) (Database Management system), as well as a client that transmits data towards the SmartAgro central cloud. The communication with the SmartAgro cloud will be based on a publish/subscribe mechanism (MQTT broker), which will be implemented within the cloud platform. This component is aimed to assure data persistence and to decrease the latency in some scenarios, when only a group of parcels is targeted for off-line tasks or processing.
- **Arrowhead Local Cloud:** After the MQTT broker, a Data Transformation Engine will extract and format the heterogeneous sensor data into structured data. Here, a new decision support component will take decisions based on the data from all parcels, previously processed and analyzed. In the Arrowhead Local Cloud, device and resource management services will be implemented and notifications and alerts will be sent when the parameters are outside the proper range or when there are signaled devices failure and faults.

Currently the project is in the stage of testing and implementing Arrowhead tools and its efficiency is going to be compared to the previous solutions in the context of a new and innovative architecture for the telemetry system.

## 5. New Research Lines, Open Issues, and Challenges

We studied several use cases pertaining to using the Arrowhead Framework in smart cities and smart agriculture. The applications, architectures, and performance of those contributions have been reviewed and illustrated. This opens several research directions and open issues that are discussed in the following paragraphs.

### 5.1. Interoperability

Even today, there is a plethora of proprietary IoT systems that use proprietary data formats. It is necessary, in order to enable the future SoS concept, that systems communicate successfully between them and are plug’n play systems. SoS actually pose significant technical challenges in terms of information interoperability that require overcoming conceptual and technological barriers. Even today, within many modern ICT systems, interoperability is not seen as a strong requirement within their design. This leads to limited interoperability between systems and a high cost associated with the alignment of the systems.

Using Arrowhead as a core framework might enable the achievement of interoperability. Currently Arrowhead provides an interoperability core system supporting protocol and encoding translation with a wide variety of protocols, conditioned by agricultural use case requirements.

### 5.2. Scalability

All systems must support adding new sensors, whether the same or different than the already existing types. Moreover, it is necessary to be able to deploy a system or a SoS on a wider scale starting from, e.g., an existing pilot. A necessary requirement is that service and application developers do not develop from scratch but instead reuse and adapt existing components to their needs.

As Arrowhead Framework is designed to work with local clouds, this can enable a scalable approach where all local clouds have the same type of configuration, and automated systems can intrinsically scale.

### 5.3. Ontology and Semantic Data

The key challenge of integrating different agricultural IoT systems is how to deal with the semantic heterogeneity of these multiple information resources. Therefore, one requirement includes an ontology-based approach to describe and extract the semantics of agriculture IoT objects and a mechanism for sharing and reusing agricultural specific knowledge. While for other disciplines (e.g., eHealth) ontologies have already been well established, for agriculture little efforts have been made especially since it might not seem necessary currently.

The problem of semantics translation is very complex, but Arrowhead Framework is designed to tackle this. Now, there are experimental core systems that are to be further matured by such logical entities as Consumer—CodeGeneration, LegacyIntegration, ModbusTCP, and SemanticsTranslator. On top of this, specific ontologies for agriculture can be built.

### 5.4. Agricultural Service Discovery and Provision

We see this as more and more prevalent in the future. In an agricultural infrastructure, where parts of an application are divided into several pieces (for example: Agri-services in a microservice infrastructure), there is usually the need to handle the client requests to a service by means of a load balancer. Therefore, service discovery will be an essential part of future smart agriculture, not only by means of simple pointing to a correct address of a service, but also by enabling advanced mechanisms such as service health checks or even self-healing.

Another concept, the agricultural service provision, will be equally essential as the agricultural IoT services can be provisioned when connecting new devices by discovering and configuring the relevant endpoint and identifying the service capabilities offered by the platform. All this has the goal to offer to the end-user a fast and ready-to-use configuration of the system without the need for any specific ICT knowledge.

As mentioned, the Arrowhead Framework contains the mandatory ServiceRegistry system, which enables systems within the local cloud to publish their services. However, it has to transition to an approach where the core system automatically discovers services contained in systems in the local cloud. On the other hand, if the systems in the local cloud do not have any, the AF can automatically provision it with the right services based on the identification of the system type.

### 5.5. Standards

Currently there are no existing established smart farming standards, although many un-coordinated initiatives exist. There is a great need for standardized IoT agricultural data formats, IoT transmission protocols, and IoT agricultural specific reference architectures. Standards are needed to ensure the safety of agricultural platforms and devices, to ensure that agricultural products and materials are specifically made for their purpose, and to promote the interoperability of agricultural products and services. The existence of standards will enable the surpassing of all the aforementioned challenges (interoperability, scalability, ontologies and service discovery and provision.)

Arrowhead is particularly suited to standardization, as its complete reliance on standardized formats, protocols, and technologies will influence standards and frameworks into being adopted.

### 5.6. User-friendliness and Visualization

There are many initiatives presently for nonskilled agriculture stakeholders, however, we have to point out that for an end-user it is highly important to not only have relevant data in an at-a-glance, easy to understand visual format, but also to be offered already insight and decision support based on data analytics. Future smart agriculture SoSs should be highly adapted to the different way of processing/analyzing data according to who is the end-user: For example, a farmer or cooperative (in the first case it means a friendly and relatively punctual analysis, in the second case, a more complex analysis).

## 6. Conclusions

Arrowhead Framework was designed with the main purpose to enable interoperability between IoT components and to implement SoS from separate IoT systems. The area of applicability for Arrowhead Framework and Tools expands from basic engineering systems up to smart applications and systems including smart grid, smart energy, smart cities, smart constructions, smart agriculture, etc. Basic advantages of Arrowhead derive from SOA capabilities: Scalability, large interconnectivity between different IoT devices, efficiency in resource allocation and consumption.

In smart cities development, Arrowhead proves efficient in the components reusability utility system, improvement energy consumption, etc. Yet, solutions related to communication security in terms of data protection are still to be developed.

Smart agriculture requires a high initial investment, efficient farming tools, and skilled and knowledgeable farmers or growers, among others. Moreover, the cloud-based platform is a breakthrough aspect of smart agriculture which is not common in the farming industry. Major factors restraining the growth of the smart agriculture market are high cost and limited technical knowledge and skills of farmers.

Smart agriculture use-cases require multiple data resources integration achieved with an increased number of sensors. News systems based on innovative solution will rely on application of the necessary technologies to allow the generation of a technological platform with analytical capacity, both in real time and pseudo real time, providing predictive and self-learning capabilities.

Typically, the data registered by sensors must be carried out by technologies linked to IoT. To this end, data capture buses will be implemented in real time to allow the disambiguation of the input data. This will be achieved by applying the semantic interpretation to the data and allowing its linkage to the analysis entities to be defined. To achieve this capture, protocols such as MQTT, Advanced Message Queuing Protocol (AMQP), or Java Message Service (JMS) will be applied to ensure the entry of messages in the system. This information will be routed by the messaging brokers. This messaging brokers in combination with the Real-Time Processing Systems (CEP) will allow the analysis through the combination of different input variables.

When designing a smart agriculture solution with Arrowhead Framework architecture other innovative aspects include Smart Farming management, Cloud storage and Platform as a Service (PaaS), IoT ready, multiprotocol for connectivity, cybersecurity, and Traceability.

Finally, analyzing the advantages of Arrowhead Framework, the SmartAgro project team decided to propose a novel Smart Agriculture architecture based on Arrowhead network. Being currently under testing, the results of the tests performed will be emphasized in a future work.

## Figures and Tables

**Table 1:** The information of sweat sample Point-of-Care Testing (POCT) device [12].

Theory	Detect Target	System Components	Time	Limit of Detection (LOD)	Disease
**Fluorescence**	Cl^−^	LED, battery, filter, 3D-print case, chloride sensors	–	0.8–200 mM	Cystic fibrosis
**Fluorescence**	Cl^−^, Na^+^, Zn^2+^	Dark box with phone holder, sweat patch, emission/excitation filer	~20 min	5–100 mM	Cystic fibrosis
**Electrochemical**	Glucose	Three-electrode system, Bluetooth chipset, lithium battery	30 min	15 μM	Diabetes
**Electrochemical**	Glucose, Lactate, Na^+^, K^+^, Temperature	Battery, sensor array, electrode arrays, Bluetooth transceiver microcontroller	~20 min	–	Hyponatremia, Hypokalemia
**Electrochemical**	Cl^−^, Na^+^, Glucose	Microcontroller, sensor array, electrode arrays, Bluetooth transceiver, battery	20–25 min	–	Cystic fibrosis

**Table 2:** Use-cases and practical examples for Arrowhead Framework [42].

Use-Case	Goals	Examples
SoS engineering of IoT devices (including energy domain)	Engineering development of IoT systems, including edge IoT devices and cloud-based integration platform [43].	[39,43,44]
Production support, energy efficiency, task management, data analytics and smart maintenance [43]	Improvement of different processes (manufacturing, maintenance, and engineering applications) including semiconductor front end and facility core processes.	[45,46]
Flexible data acquisition system	Ensuring interoperability between different data acquisition devices in production area.	[47]
Local clouds autonomous configuration	Development of local automation clouds.	[48,49,50,51]
Deployment and configuration of SoS systems	QoS improvement for SoS integration.	[52,53]
Semiconductor industry	Fast and reliable solutions for semiconductor industry.	
Automation tools merge with product lifecycle tools	Improvement of new IoT systems performances and efficiency.	

**Table 3:** Comprehensive 802.15.4g network testing results [57].

**Data rates (Kbps)**		**Throughput (KB)**			**Delay (min)**		
		200	400	800	200	400	800
**SNR (dB)**		9	15	21	9	15	21
**No. of poles**	1000	779.67	958.97	1157	9	7	6
		899.67	1778.7	3477.72	8	4	2
	5000	149.88	176.44	192.99	48	40	37
		124.03	210.34	411.25	58	34	17
	10,000	74.57	87.35	94.53	96	82	76
		58.64	80.79	155.99	122	89	46
